# Leveraging healthcare utilization to explore outcomes from musculoskeletal disorders: methodology for defining relevant variables from a health services data repository

**DOI:** 10.1186/s12911-018-0588-8

**Published:** 2018-01-31

**Authors:** Daniel I. Rhon, Derek Clewley, Jodi L. Young, Charles D. Sissel, Chad E. Cook

**Affiliations:** 1 Center for the Intrepid, Brooke Army Medical Center, 3551 Roger Brooke Drive, San Antonio, TX 78234 USA; 2 Baylor University, 3630 Stanley Road, Bldg 2841, Suite 1301; Joint Base San Antonio - Fort Sam Houston, San Antonio, TX 78234 USA; 3 Division of Physical Therapy, Department of Orthopedics, Duke University, 2200 W. Main Street, Durham, NC 27701 USA; 4 Department of Physical Therapy, Arizona School of Health Sciences, 5850 E. Still Circle, Mesa, AZ 85206 USA; 5 Headquarters, U.S. Army Medical Command, Analysis & Evaluation Division, 3630 Stanley Road; Joint Base San Antonio - Fort Sam Houston, San Antonio, TX 78234 USA

**Keywords:** Database research, hip, arthroscopic surgery, healthcare utilization

## Abstract

**Background:**

Large healthcare databases, with their ability to collect many variables from daily medical practice, greatly enable health services research. These longitudinal databases provide large cohorts and longitudinal time frames, allowing for highly pragmatic assessment of healthcare delivery. The purpose of this paper is to discuss the methodology related to the use of the United States Military Health System Data Repository (MDR) for longitudinal assessment of musculoskeletal clinical outcomes, as well as address challenges of using this data for outcomes research.

**Methods:**

The Military Health System manages care for approximately 10 million beneficiaries worldwide. Multiple data sources pour into the MDR from multiple levels of care (inpatient, outpatient, military or civilian facility, combat theater, etc.) at the individual patient level. To provide meaningful and descriptive coding for longitudinal analysis, specific coding for timing and type of care, procedures, medications, and provider type must be performed. Assumptions often made in clinical trials do not apply to these cohorts, requiring additional steps in data preparation to reduce risk of bias. The MDR has a robust system in place to validate the quality and accuracy of its data, reducing risk of analytic error. Details for making this data suitable for analysis of longitudinal orthopaedic outcomes are provided.

**Results:**

Although some limitations exist, proper preparation and understanding of the data can limit bias, and allow for robust and meaningful analyses. There is the potential for strong precision, as well as the ability to collect a wide range of variables in very large groups of patients otherwise not captured in traditional clinical trials. This approach contributes to the improved understanding of the accessibility, quality, and cost of care for those with orthopaedic conditions.

**Conclusion:**

The MDR provides a robust pool of longitudinal healthcare data at the person-level. The benefits of using the MDR database appear to outweigh the limitations.

## Background

Health services research (HSR) is widely defined as “the multidisciplinary investigation of how social factors, financing systems, organizational structures and process, health technologies, and personal behaviors affect access to healthcare, the quality and cost of healthcare, and ultimately our health and well-being [1].” In simpler terms, HSR aims to study access to care, quality of care and the cost associated with care. Large databases can collect all of this information, greatly enhancing HSR.

Big data in healthcare is defined as “high-volume, high-velocity and/or high-variety information assets that demand cost-effective, innovative forms of information-processing that enable enhanced insight, decision making and process automation [2, 3].” Many healthcare organizations already collect this “big data” in order to remain compliant with regulatory agencies, drive better business practice, maintain high standards for patient care, and perform efficient and effective record keeping [4]. Data is captured from many sources at a real time rapid pace known as velocity. This velocity, along with variety of data, creates a significant challenge for cleansing and analyzing the data. In fact, these datasets can be so large, overwhelming, and complex that traditional software and hardware are insufficient [2].

Healthcare databases are valuable for assessing business practice and research on healthcare outcomes. Clinical trials are very costly, often include patients that are lost to follow-up, and have relatively shorter timeframes for follow-up. Using healthcare databases for research comes with its own set of limitations; however, the cohorts can be substantially larger with much longer follow-up. In addition, healthcare utilization allows for highly pragmatic research. While explanatory trials are designed to determine the effects of an intervention under ideal conditions, pragmatic research aims to assess the effects of an intervention under the usual setting in which it is applied [5]. Clinicians looking to published research for guidance with decision-making are best served by studies with a pragmatic design [6–8]; however, the overwhelming majority of medical studies to date have used explanatory designs [7]. Specifically with regards to outcomes, highly pragmatic research has been defined as having no formal follow-up, but instead uses databases for the detection of outcomes [5].

Healthcare databases can be a rich source of data for understanding care pathways for orthopaedic conditions. They can provide cross-sectional and longitudinal interactions based on time and healthcare events. We recently identified a cohort of patients that received arthroscopic hip surgery within the United States Military Health System (MHS) over a 10 year period. The purpose of this paper is to use this surgical cohort as an example to describe the methodology employed to create meaningful data from large healthcare databases, and discuss relevant data considerations. In particular, we will discuss the methods utilized to address many of the ongoing challenges associated with use of big data, including: 1) sourcing data; 2) organizing data for clinical relevance [9]; 3) coding in a meaningful and descriptive way [10]; 4) handling missing values [11–14]; 5) reporting outcomes; 6) assuring the clinical veracity of the data [10, 15]; and 7) reducing risks of analytic errors [16].

## Construction and content

### Data Sourcing

#### Origination of Data

The Defense Health Agency (DHA) supports the delivery of healthcare to approximately 10 million beneficiaries of the MHS through the TRICARE Health Plan. The Decision Support Division of the DHA is responsible for managing the specifications of the MHS Data Repository (MDR), which serves as the centralized repository for all DHA corporate healthcare data fed from a worldwide network of more than 260 Department of Defense (DoD) healthcare facilities and a few non-DoD entities. The MDR collects an incredible volume of data from around the world every single day. It captures, archives, validates, and merges data from over three dozen independent healthcare databases, and then quickly integrates and distributes it in a way that can be used for clinical and business decision support. Data capture comes with unique challenges in that it has to account for military healthcare encounters all around the world, to include austere environments during training, combat and humanitarian operations overseas, as well as care that occurs on naval vessels such as aircraft carriers and submarines. This repository includes records of every single person-level interaction for healthcare where the TRICARE Health Plan is the payer, both inpatient and outpatient, and either in a civilian or DoD facility. For all visits within military treatment facilities (MTF), these person-level visits include variables such as vital signs, body mass index, tobacco usage, inpatient and outpatient medications, and chemistry lab results. For records from fiscal year 2000 and forward, the MDR contains a unique person identifier allowing person-level files to be linked across data sources, and is considered the most reliable source for MHS data. It also contains accounting data for each MTF, beneficiary, and staffing. The MDR data dictionary is publicly available and can be accessed at the DHA’s health.mil website. The structure of the data is Serial-Attached Small Computer System Interface.

#### Individual Data Files that Feed into MDR

Over 38 unique data files from hundreds of data sources feed into the MDR daily from around the world, and files from each source are aggregated by fiscal year. Data across multiple files are connected based on a unique person identifier. The following are the data files from the MDR we found most relevant to the study of musculoskeletal conditions, and stored in the MDR in Statistical Analysis System (SAS) format (except for pharmacy data, which is stored in text format):

1. Standard Inpatient Data Record (SIDR): This information includes diagnosis and procedure codes, length of stay, cost and relative weighted products (analogous to Relative Value Unit - RVU) for each episode of care, and departments rendering care for every inpatient hospital admission that takes place within a MTF.
2. Comprehensive Ambulatory/Professional Encounter Record (CAPER): Each unique observation within the CAPER file represents one ambulatory outpatient encounter or professional service taking place within a MTF. The information captured includes diagnosis and procedure codes, cost and RVUs for each episode of care, and provider type and departments rendering care.
3. TRICARE Encounter Data - Institutional (TED-I): The information captured in the TED-I includes diagnosis and procedure codes, length of stay, actual cost paid by TRICARE and relative weighted products (analogous to RVUs) for each episode of care, and departments rendering care for every civilian inpatient hospital or institution-based home healthcare encounter.
4. TRICARE Encounter Data - Non-Institutional (TED-NI): Each unique observation within the TED-NI file represents one line item on a claim, taking place in any civilian setting to include professional inpatient services, but not care related to hospital admissions. One medical visit can result in multiple line items on a claim, or even multiple claims. This includes any ambulatory outpatient encounters, pharmacy, radiology and laboratory tests, ambulance services, and medical supply related encounters. The information captured includes diagnosis and procedure codes, cost and RVUs for each episode of care, and provider type and departments rendering care.
5. Pharmacy Data Transaction Service (PDTS): Each unique observation with the PDTS file represents an outpatient prescription filled for a MHS beneficiary, whether at a MTF facility, retail pharmacy in the United States, or through the mail-order program. The information captured includes generic drug name, therapeutic class codes, date of prescription, cost, and the days’ supply of each prescription. The PDTS file does not include prescriptions from inpatient settings or those from civilian pharmacies outside of the United States.
6. Ancillary Care: The ancillary care file captures all completed laboratory and radiology procedures that take place in a laboratory or radiology department within a MTF. Purchased ancillary care occurring in civilian settings is captured in the TED-NI file. The information includes procedure codes, accession numbers linking the procedure to the visit where it was ordered, indication of whether the procedure came from an inpatient encounter, and date of procedure.

#### Merging Individual Data Elements and Requesting Data from MDR

Data are pulled across year and across files through linking of unique person identifiers common to all files. This allows for longitudinal assessment of a large variety of healthcare variables at the single person-level. Utilization of data for research purposes require several steps. First a Data Sharing Agreement must be approved by the DHA Privacy Board, and second an analyst with proper training and access to the MDR must be identified.

#### Data Cleaning Procedures by DHA

The DHA utilizes a robust method for addressing errors in data. Data from MTFs are transmitted from the electronic medical records to the MDR daily, and the MDR processes the data weekly. The data at this point are raw (unprocessed). The master data file is updated monthly, and at this point, both new processed data and updates to previous data are added to the master file. Requests for healthcare utilization through a given time period wait a minimum of 90 days after the event of interest in order to ensure the data are captured, processed, and updated in the master data file. Any files containing a blank encounter identification number, International Classification of Diseases (ICD) or Current Procedural Terminology (CPT) code, or with a visit count of zero, are written to an error file. The exception are CAPER files that are not required to have a CPT code for every visit. These encounters continue to go through an extensive validation process across multiple sources to in order to fill in the missing variables. This process is explained in detail via documentation publically available online [17]. Once an encounter is validated (not raw), it will be uploaded into the master file in a subsequent update.

For purchased care occurring outside of a MTF, the claims are initially submitted by the Managed Care Support Contractors for payment by TRICARE. After the payment has been processed, the records are uploaded to TRICARE’s TED system on a monthly basis, and then processed and stored as a final dataset in the MDR. Data from the MDR are added to the TED encounters as they are processed to improve the utility of the records.

##### Handling of Missing Values

Missing data values are a critical challenge when dealing with large datasets. Missing data values can be defined as missing completely at random (MCAR), missing at random (MAR), or not missing at random (NMAR). The MDR utilizes multiple checkpoints to improve data quality and consequently has minimal missing values. As an example, descriptive variables such as age, gender, surgery type, rank, and total post-surgical healthcare visits and costs have almost zero instances of missing values. In the rare cases where missing values existed, we used Little’s test of MCAR to investigate the relationship between the missingness of the data and any values, observed or missing after [18], including those variables we planned to use in future analyses. Little’s MCAR test was not significant, suggesting that the missing data points are a random subset of the data and that there is no systematic process resulting in some data more likely to be missing than others. In all cases analyzed, non-significant findings were present, suggesting that data were missing at random.

We used a chains equation multiple imputation which operates under the assumption that given the variables used in the imputation procedure, the missing data are missing at random. This means the probability that a value is missing depends only on observed rather than unobserved values [19]. The missing values were imputed based on the observed values for a given individual and the relations observed in the data for other participants, using both predictor and imputed codes. We also performed a sensitivity analysis to determine if the newly created imputed dataset yielded different results from the original dataset. The sensitivity analysis was performed by comparing the average of the estimated regression coefficients (Bmissing) to the reference values from the fully observed data set (Bfull). We calculated a percentage difference between the mean of the estimated coefficients through the following calculation: (Bfull - Bmissing/Bfull) X 100%. The magnitude of the relative bias was graded using the parameters provided by Henry et al. [20], and included: 0% to 5%=negligible; 5.1% to 10%=minimal; 10.1% to 20%=moderate; 20.1% to 30%=heavy; and >30.1 as severe. In all cases, the differences in estimated coefficients were <10%.

##### Assuring the Clinical Veracity of the Data

Rarely do datasets and clinical trials reflect the same intent or findings. Veracity of data, or the uncertainty of the data included in the analyses, reflects the ability of the database to be truly representative of what happened at a clinical level [21]. In order for the data to have veracity, the findings must indeed tell a story that is meaningful. Health services research examines how people get access to healthcare, the cost of that care, and what happens to patients as a result of this care [1]. The MDR allows one to investigate costs for specific interventions, identify those who have access to care, and capture downstream care (or a lack of care). Further, because comorbidities, multiple provider types, other forms of care, and other elements are included in the dataset, investigation of the interrelationships of the data has merit.

##### Reducing Risks of Analytic Error

Although the MDR is a robust database, it is important to limit assumptions that may be pertinent in a clinical trial. No matter what adjustments are used, it is not safe to assume that baseline comparisons of groups who received two different care pathways or intervention types are actually similar. When modeling, it is important to investigate for incidental endogeneity and multicollinearity of every predictors for all occasions. This requires controlling for covariates using the risk adjustment measures that we have described, along with other confounding elements identified. Outlier and sensitivity analyses should be run, and the approach adjusted as needed. Reporting confidence intervals of the data for all models, and providing full disclosure in all statistical analyses is important.

## Utility

### Organization of Data for Clinical Relevance

#### Normalization of Data

Using healthcare utilization to determine study eligibility comes with several challenges. As with similar planning for other study designs, homogeneity of subjects is important in order to derive generalizable conclusions. While there are many factors to consider, three are key when initially creating a cohort from a healthcare database: inclusion criteria, exclusion criteria, and database eligibility. The use of ICD codes are associated with every encounter and enable filtering of care based on diagnosis. Finally, confirmation that patients are eligible beneficiaries during the entire period of surveillance is important. In the MDR, this is done through the Defense Enrollment Eligibility and Reporting System (DEERS) file.

#### Selection of Variables

The following is a description of the methodologies utilized in the data extraction, derivation, and definition of variables from the MDR. The variables represent healthcare events captured during a 36-month period of time for patients undergoing hip surgery.

**Fig. 1:**
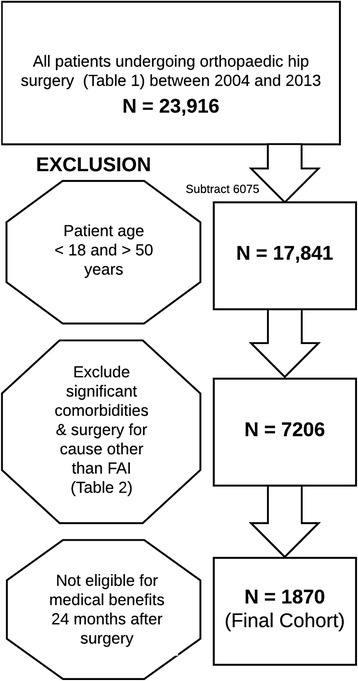
Cohort selection process

For our analyses, we targeted all patients ages 18 to 50 who had undergone hip surgery for femoroacetabular impingement (FAI). Because a dedicated diagnosis code for FAI does not exist, we identified common surgical procedure codes for this condition as inclusion variables (Table 1). We then excluded any non-FAI conditions that might also receive this same surgical procedure (Table 2). We did not exclude persons with these codes that occurred after the surgery, as they may have had a relationship to the surgical procedure. Finally, we had to exclude everyone that was not continuously eligible as a TRICARE beneficiary for the entire 12 months before and 24 months after the surgical procedure (Fig. 1).

**Table 1:** Current Procedural Terminology (CPT) Codes for Arthroscopic Hip Surgery

CPT Description (Arthroscopic)	CPT Code
29914	Arthroscopy, hip, surgical; with femoroplasty (ie, treatment of cam lesion)
29915	Arthroscopy, hip, surgical; with acetabuloplasty (ie, treatment of pincer lesion)
29916	Arthroscopy, hip, surgical; with labral repair
29862	Arthroscopy, hip, surgical; with debridement/shaving

**Table 2:** Excluded Non-FAI Conditions that might receive same surgical procedure

Diagnosis	ICD-9 Codes
Hip Osteoarthritis	715.15, 715.25, 715.35, 715.95
Avascular necrosis of the hip	733.42
Hip Fracture	820, 821
Osteomyelitis of the hip	730.85
Malignant neoplasm of the pelvis, hip, or lower extremity	170.6, 170.7, 171.3
Other hip arthritic condition	714.0, 711.05

*ICD* International Classification of Diseases, 9th Edition

#### Meaningful and Descriptive Coding

One of the most notable challenges when working with large datasets is the creation of meaningful and appropriately descriptive codes for quantitative use. Codes for timing and types of procedures, provider type, and use of accessory care options (e.g., medications) are also available and essential. These include surgery, radiology, injection, and physical rehabilitation codes. Identifying providers who deliver different types of care can provide information about optimal pathways of care. This was possible because all medical encounters from the MDR data provided the Healthcare Provider Taxonomy Code, as established by the Health Insurance Portability and Accountability Act (HIPAA). This code identifies the provider type, such as “physical therapist,” “nurse practitioner,” “family physician,” etc. These codes can be found on the Centers for Medicare and Medicaid Services government website. The MDR database also provides a product line code, which indicates the service department where care took place (eg. primary care, orthopaedics, physical therapy, etc.) for direct care (CAPERS and SIDR), but is based on the provider specialty for purchased care (TED). All prescriptions provided to patients during the entire period of surveillance were also abstracted, therapeutic class codes for opiate drugs (280808 and 280812) were flagged. The MDR also provided the date and total days’ supply of the prescription.

#### Access and Timing to Care

A key component of health services research is access and timing to care [22]. Because the MHS is a closed single-payer system, and healthcare payments are standardized among recipients, access to care due to personal cost is a lesser issue than in civilian care. Within the MDR, timing of care is available, as every medical visit documented has a date of encounter. Past studies have found that the timing of care can reduce downstream costs and unnecessary procedures [23, 24]. Some providers may also utilize more medical resources than others. For example, utilization of healthcare and associated costs varies widely based on the type of provider seen. Patients with low back pain who seek care from a physical therapist often have fewer radiographs and surgeries [23, 24]. In contrast, those who are seen in physiatry are at an increased risk for radiographs, advanced imaging, invasive injections, surgery and other healthcare costs [25]. Chiropractic care is associated with decreased advanced imaging or surgeon visits, similar to physical therapy, but with an increased length of time in intervention [26]. Some evidence also suggests that seeing multiple providers leads to higher costs of care [23]. Lastly, there are often regional differences in the type of services individuals receive. In the United States for example, the Northeast region has the fewest surgeries and injections, and the Midwest and South have the highest [18, 24].

#### Comorbidities

An essential element of data for use in health services research is the ability to accurately reflect the health condition, at a person-level, for each individual. Historically, researchers have used comorbidities to describe person-level conditions that could mediate outcomes, and control for the influence of these variables on the targeted outcomes during modeling. These are important to consider, as many analyses related to outcomes should adjust for these conditions and their influence on prognosis. Depending on the analysis, conditions such as the presence of malignant neoplasms can be identified; however, in most cases these individuals are excluded from the cohort. We identified a list of medical comorbidities within the MDR that we found to have a significant association with orthopaedic injury and surgical outcomes (Table 3). The MDR validation system also includes identification and flagging of industry-related codes related to patient disease profile. These include the Center for Medicare and Medicaid Services (CMS) Hierarchical Coexisting Conditions (HCC) risk score which calculates a risk score for adjusted comparisons of patients with various disease profiles and the Agency for Healthcare Research and Quality (AHRQ) Chronic Condition Indicator (CCI), which categorizes conditions into chronic and not chronic.

**Table 3:** Comorbidities, Operational Definitions, and Support for Use

Comorbidity	Codes to Identify	Support for Use
Sleep Disorders	ICD-9 codes of: *Insomnia:* 307.41, 307.42, 327.00, 327.01, 780.52, v69.4	Sleep disorders are a specific risk factor for increased health seeking in pain populations [29], and associated with higher rates of consulting for low back pain [30]. Poor sleep induces generalized hyperalgesia, increases anxiety, and affects one’s ability to regulate cortisol in response to stress [31–33]. Specifically, severity of insomnia has been correlated with pain intensity in patients with chronic musculoskeletal pain [34].
	*Sleep-related breathing disorders:* 320.20, 327.21, 327.22, 327.23, 327.24, 327.25, 327.26, 327.27, 327.29, 768.04, 770.81	
	*Hypersomnias* 307.44, 327.10, 327.11, 327.12, 327.13, 327.14, 327.15, 347.00, 347.01, 347.10, 780.54	
	*Circadian rhythm sleep disorders:* 327.31, 327.32, 327.33, 327.34, 327.35, 327.36, 327.37, 327.39	
	Parasomnias: 307.46, 307.47, 327.41, 327.42, 327.43, 227.4, 300.15, 327.44, 327.49, 368.16, 788.36	
	*Sleep-related movement disorders:* 327.51, 327.52, 327.53, 327.59, 333.49	
Mental Health Disorders	ICD-9 codes of 295.xx, 296.xx, 297.x, 298.x, 300.xx, 301.xx, 307.8x, 308.xx, 309.xx, 311.xx, v11.x, v15.52, v40.9	Mental health disorders have been shown to impact overall pain and function in patients with musculoskeletal disorders. Depression and anxiety in patients with chronic musculoskeletal pain is associated with higher pain and disability levels, as well as a worse health-related quality of life [30]. Mental health comorbidities are associated with development of chronic disease/disorder, higher overall healthcare utilization and consequently higher costs [31], and contribute to overall disability levels. Post-traumatic stress disorder (PTSD) is ICD-9 code 309.81, specifically.
Substance Abuse	ICD-9 codes of 303.xx, 304.xx, 305.xx	Substance use includes unhealthy dependencies on prescription drugs or alcohol as well as personal history of tobacco use. These dependencies can affect outcomes after musculoskeletal pain or injury in many ways. Opiate use is associated with higher rates of medical visits and healthcare utilization [31]. In those who have chronic musculoskeletal disorders and take higher dosages of opioids long term, there is greater overall healthcare utilization and lower rates of return to work or job retention [33]. Significant use of alcohol for pain relief in people who have chronic musculoskeletal pain can affect sleep and depressive symptoms, which ultimately can increase pain levels, not decrease them [34]. In one population of people with chronic non-cancer pain who were taking opioids, risky levels of drinking alcohol were related to poorer pain outcomes [35]. Smoking is adversely related to functional outcomes after injury, independent of the nature or severity of injury [35]. It is an independent risk factor across a variety of populations and conditions [36, 37], and negatively influences healing after orthopaedic surgeries [38]. It has deleterious effects on peak bone mass [39], bone mineral density, [40] bone healing [41], and wound healing [42], as well as many other general complications [30, 43]. Chronic smokers are also at higher risk for developing muscle pain [44].
Tobacco Use	ICD-9 code of V15.82	Smoking is adversely related to functional outcomes after injury, independent of the nature or severity of injury [35]. It is an independent risk factor across a variety of populations and conditions [36, 37, 45]^,^ and negatively influences healing after orthopaedic surgeries [38, 46]. It has deleterious effects on peak bone mass [39], bone mineral density [40], bone healing [41], and wound healing [42, 47], as well as many other general complications [48]. Chronic smokers are also at higher risk for developing muscle pain [44].
Metabolic Syndromes (obesity, diabetes, etc.)	ICD-9 codes of 249.xx, 250.xx, 255.0, 272.0, 272.2, 272.4, 278.00-278.03, 278.8, v85.30-v85.39, v85.41-v85.45	Metabolic syndromes affect orthopaedic outcomes. Obesity increases the risk for musculoskeletal disease in not only bones and joints, but also soft tissues [49]. In those with obesity related disease, direct healthcare costs, including medication use, inpatient and outpatient visits, are high [50]. Diabetes can adversely affect wound healing, potentially leading to non-healing wounds that cost upwards of $3 billion per year [51].
Chronic Pain	ICD-9 codes of 338.2x, 338.3, 338.4, 729.1, 780.71, 780.79ICD-9 codes of 338.2x, 338.3, 338.4, 729.1, 780.71, 780.79	Chronic pain has been defined as persistent or recurrent pain lasting longer than 3 months [52], and present after orthopaedic surgery [53]. Poor post-surgical outcomes including pain and patient satisfaction have been associated with pre-surgical chronic pain [54]. Fibromyalgia is a condition characterized by widespread body pain, fatigue, poor sleep, and depression. Characteristics of fibromyalgia have been shown to be predictive of poor post-surgical outcomes [55].
Cardiovascular Diseases	ICD-9 codes of 348.2, 401.xx-405.xx, 410.xx-414.xx, 420.xx-429.xx	Cardiovascular conditions are a group of disorders of the heart and blood vessels. Heart disease has been identified as a comorbidity that predicts poor pain outcomes after total knee and total hip arthroplasty [56]. Congestive heart failure has been shown to be a predictor for total hip revision surgery within 12 months of the original total hip arthroplasty [57]. Cardiovascular disease is a preoperative predictor of poor postoperative subjective outcome for lumbar spinal stenosis surgery [58].
Systemic Arthropathies	ICD-9 codes of 099.3, 274.xx, 696.0-696.8, 710.0, 710.2, 711.15, 711.25, 711.35, 711.45, 711.55, 711.65, 711.75, 711.85, 712.15, 712.25, 712.35, 712.85, 712.95, 714.0, 714.1, 714.2, 714.4, 714.89, 716.25, 718.55,719.35, 720.0, 720.81, 720.89, 720.9, 725, 729.0	There are a number of systemic conditions that can cause pain in multiple joints. Some examples of systemic arthropathies are: ankylosing spondylitis, rheumatoid arthritis, psoriatic arthritis, and juvenile rheumatic arthritis. These comorbidities are associated with increased post surgical pain, decreased function, and reduced quality of life [57]. The economic and societal burden of systemic arthropathies is significant. Healthcare expenditures for those with rheumatoid arthritis are over three times greater than those without rheumatoid arthritis. Adjusted for comorbidities, incremental healthcare expenditures are over $2000 annually [59]. Opioid prescriptions for arthritis related pain has dramatically increased [60], which also drive up healthcare costs.

.xx = wildcard variable, any integer after the preceding number is included

#### Creation of a Risk Adjustment Code Based on Comorbidity Status

HCCs are proprietary formulas, typically used for Medicare populations, and use data to prospectively estimate predicted costs for enrolled individuals during a follow-up year of coverage. These predictions are based on demographic information and major medical conditions documented from patient encounters in a previous 12-month period. HCCs provide a cost-related risk to the overall outcome since these define which codes are related to the highest costs for care. We used a regression based risk adjustment mechanism to control risks of higher costs based on selected health conditions that is similar to HCC. Our weighted risk adjustment variables were built on the presence of comorbidities and the weighted influence of those comorbidities, in single or in combination, on the outcomes of costs and visits.

### Outcomes Reporting

Although the MDR lacks self-reported outcomes data, the database does include costs, visit utilization for each provider type, care process (e.g., physical therapy, medications), use of medications, and location (MTF or in-network provider). Costs are sub-categorized by total costs of all healthcare interventions (which in our cohort included hip and non-hip related costs) and total costs of hip related healthcare interventions. Costs for provider and treatment domain are each categorized. Purchased care reflects what the TRICARE Health Plan actually paid for the service. If patients have other health insurance (OHI) plans, then the payment by TRICARE may be decreased in some cases. This is most relevant in patients above the age of 65 with Medicare. Approximately 2 million TRICARE beneficiaries (~20%) have OHI. However, in the cohort with the demographic we sampled (ages 18 to 50), the number of patients with OHI was less than 3%. For direct care, the “Full Cost” of the encounter is broken down into elements which allow the analysts to identify unique distributions and allocations of care. For research purposes, we used the “Full Cost” variable that includes all components (Table 4).

**Table 4:** “Full Cost” elements for care that takes place in a Military Treatment Facility (Direct Care)

Full Cost Subcomponent Variable	Description
FCCLNSAL	Clinician salary portion of full cost
FCLAB	Laboratory portion of full cost
FCOST1	Full cost for the E&M APG
FCOST2	Full cost for the medical APG
FCOST3	Full cost for Procedure 1 APG
FCOST4	Full cost for Procedure 2 APG
FCOST5	Full cost for Procedure 3 APG
FCOST6	Full cost for Procedure 4 APG
FCOTHANC	Other ancillary portion of full cost (minus materials)
FCOTHLBR	Other labor portion of full cost
FCPROFSAL	Professional salary portion of full cost
FCRAD	Radiology portion of full cost
FCRX	Pharmacy portion of full cost (minus materials)
FCSUP	Support portion of full cost

*APG* Ambulatory Patient Group, *E&M* Evaluation and Management Coding in support of medical billing

Visits are structured by provider, similarly to costs. Each visit is uniquely captured and dated in the MDR, allowing for analyses of trends of care. Further, because we plan to explore the modeling of predictors for continued opioid and other medication use, imaging use, and rehabilitation use, these variables will be included as potential outcome measures.

## Discussion

In the paper, we discussed many of the ongoing challenges associated with use of big data, such as: 1) sourcing data; 2) organizing data for clinical relevance [9]; 3) coding in a meaningful and descriptive way[10]; 4) handling missing values [11–14]; 5) reporting outcomes; 6) assuring the clinical veracity of the data [10, 15]; and 7) reducing risks of analytic errors [16].

As with all datasets, there are limitations that require discussion. The MDR does not have a patient-based health outcomes reporting mechanism, thus the ability to identify self-reported disability, quality of life, or pain-related perspectives is generally missing. Reported numeric pain scale (0 to 10) is populated for outpatient direct care visits only (CAPER), but the lack of consistency in capturing this measure makes the variable less reliable. Further, the costs used by the MDR are based on expenses captured at each military facility and RVUs at each facility. In some cases, because the facility has 45 days after the end of each month to report expenses, there is a time lag for reporting expenses and determining subsequent costs. This is done in order to capture costs accurately, and also the reason a minimum of 90 days should exist between the time of query and the through-date for data of interest. The other reason for waiting 90 days is that claims processing on the purchased care side (TED) can be delayed due to tardy claims submission. In the final MDR files available for analysis, the data are no longer raw, and cost data have been validated and updated to reflect these costs. In other words, a unit cost for each clinic is calculated using the total expenses in that clinic and the total RVUs in that clinic, which is then multiplied by the number of RVUs for each encounter. Consequently, costs are projected data based on current and past resource utilization.

The resources required to manage and maintain this repository are remarkable. The MDR is the largest and most comprehensive medical database in the United States MHS. In fiscal year 2016 alone, $36,152,000 was spent on information technology management of Defense Health Programs systems to include the MDR [27]. Although there are a limited number of persons with full level access to the MDR, the servers are often slowed down with the handling of multiple requests worldwide. Often queries have to be run at off-peak times, such as during the evening hours. It is a heavily queried and strained system. Another limitation for this particular type of research is the reconciliation of medical care covered by TRICARE and OHI. If the care occurs outside the system, and is not captured within the MDR, then an incomplete picture of the health service utilization and outcomes is portrayed for that individual. While the majority of younger families and those on active duty do not have OHI (less than 3% in the subset we analyzed), the retired military population may have higher rates of OHI as most will start a second career upon retirement from the military, which may be come with a more favorable health insurance plan. Finally, as with all data repositories, the value of the data are only as good as the care and precision taken to enter it. These data are based on claims data and diagnosis/procedure codes entered by medical staff. Variations and inaccuracies in coding nomenclature have been reported [28], and can also occur in this setting.

## Conclusion

The robust nature of the variables and large scope of data allow for many additional analyses related to outcomes. These include investigation into the utilization patterns of prescription opiate medications after surgery, the impact of opiate medication utilization on downstream costs, and their association with certain comorbidities, such as insomnia, mental health diagnoses, or chronic pain syndromes.

In summary, we feel there are more advantages to the use of the MDR than disadvantages. The most notable advantages of the MDR database include:

1. Minimal to no missing values on essential variables
2. Complete representation of all healthcare utilization before and after an index event (for those patients without OHI)
3. The ability to break down care processes by time, intensity, discipline and explore interactions of these on costs and visits
4. The ability to explore ancillary care use associated with medications, imaging use, and timing of that use
5. Strong precision of data
6. The ability to explore continuity of care management in site specific care between dedicated military facilities and in-network sites
7. Large sample sizes with clinically-reflective data that strongly represent healthcare utilization

## Abbreviations

APG: Ambulatory Patient Group
CAPER: Comprehensive Ambulatory/Professional Encounter Record
CPT: Current Procedural Terminology
DEERS: Defense Enrollment Eligibility and Reporting System
DHA: Defense Health Agency
DoD: Department of Defense
E&M: Evaluation and Management Coding
FAI: Femoroacetabular Impingement
HCC: Hierarchical Condition Categories
HIPAA: Health Insurance Portability and Accountability
HSR: Health Services Research
ICD-9: International Classification of Diseases, 9th Edition
MAR: Missing At Random
MCAR: Missing Completely At Random
MDR: Military Health System Data Repository
MHS: Military Health System
MTF: Military Treatment Facilities
OHI: Other Health Insurance
PDTS: Pharmacy Data Transaction Service
RECORD: REporting of studies Conducted using Observational Routinely-collected Data
RVU: Relative Value Unit
SAS: Statistical Analysis System
SIDR: Standard Inpatient Data Record
STROBE: Strengthening of Reporting of OBservational studies in Epidemiology
TED-I: TRICARE Encounter Data - Institutional
TED-NI: TRICARE Encounter Data - Non-Institutional
